# Effects of Multimodal Bundle with Remote Ischemic Preconditioning and Intrathecal Analgesia on Early Recovery of Estimated Glomerular Filtration Rate after Robot-Assisted Laparoscopic Partial Nephrectomy for Renal Cell Carcinoma

**DOI:** 10.3390/cancers14081985

**Published:** 2022-04-14

**Authors:** Min Suk Chae, Jung-Woo Shim, Hoon Choi, Sung Hoo Hong, Ji Youl Lee, Woohyung Jeong, Bongsung Lee, Eunji Kim, Sang Hyun Hong

**Affiliations:** 1 Department of Anesthesiology and Pain Medicine, Seoul St. Mary’s Hospital, College of Medicine, The Catholic University of Korea, Seoul 06591, Korea; shscms@gmail.com (M.S.C.); tintinshim@gmail.com (J.-W.S.); hoonie83@naver.com (H.C.); 2 Department of Urology, Seoul St. Mary’s Hospital, College of Medicine, The Catholic University of Korea, Seoul 06591, Korea; toomey@catholic.ac.kr (S.H.H.); uroljy@catholic.ac.kr (J.Y.L.); 3 Department of Anesthesiology and Pain Medicine, Bucheon St. Mary’s Hospital, College of Medicine, The Catholic University of Korea, Seoul 06591, Korea; wjddngud123@naver.com; 4 Department of Anesthesiology and Pain Medicine, Uijeongbu St. Mary’s Hospital, College of Medicine, The Catholic University of Korea, Seoul 06591, Korea; bs9811@daum.net; 5 Department of Anesthesiology and Pain Medicine, St. Vincent’s Hospital, College of Medicine, The Catholic University of Korea, Seoul 06591, Korea; eunjikk@gmail.com

**Keywords:** robot-assisted laparoscopic partial nephrectomy, remote ischemic preconditioning, intrathecal morphine block, remnant kidney function

## Abstract

**Simple Summary:**

This study suggested that robot-assisted laparoscopic partial nephrectomy (RALPN) may have benefits with regard to the preservation of renal function and few complications postoperatively in patients with renal cell carcinoma (RCC). However, a reduction in the estimated glomerular filtration rate may be unavoidable. Our results suggested that the preservation of renal function may be enhanced by combining robot-assisted nephron-sparing surgery with an intraoperative bundle strategy consisting of remote ischemic preconditioning (RIPC) and an intrathecal morphine block (ITMB), to protect against ischemia–reperfusion injury and the pain-related stress induced by renal artery clamping and surgical insults. It is important to adjust modifiable variables related to the progression of renal impairment in a timely and appropriate manner for the recovery of renal function after RALPN. Together with surgical and pharmacological methods to minimize irreversible injury, RIPC and ITMB combined bundle therapy may relieve ischemia–reperfusion- and pain-induced stress and serve as a safe and efficient method for improving renal outcomes of RALPN in patients with RCC.

**Abstract:**

We investigated the effects of multimodal combined bundle therapy, consisting of remote ischemic preconditioning (RIPC) and intrathecal morphine block (ITMB), on the early recovery of kidney function after robot-assisted laparoscopic partial nephrectomy (RALPN) in patients with renal cell carcinoma (RCC). In addition, we compared the surgical and analgesic outcomes between patients with and without bundle treatment. This prospective randomized double-blind controlled trial was performed in a cohort of 80 patients with RCC, who were divided into two groups: a bundle group (*n* = 40) and non-bundle group (*n* = 40). The primary outcome was postoperative kidney function, defined as the lowest estimated glomerular filtration rate (eGFR) on postoperative day (POD) 2. Surgical complications, pain, and length of hospital stay were assessed as secondary outcomes. The eGFR immediately after surgery was significantly lower in the bundle group compared to the preoperative baseline, but serial levels on PODs 1 and 2 and at three and six months after surgery were comparable to the preoperative baseline. The eGFR level immediately after surgery was lower in the non-bundle than bundle group, and serial levels on PODs 1 and 2 and at three months after surgery remained below the baseline. The eGFR level immediately after surgery was higher in the bundle group than in the non-bundle group. The eGFR changes immediately after surgery, and on POD 1, were smaller in the bundle than in the non-bundle group. The non-bundle group had longer hospital stays and more severe pain than the bundle group, but there were no severe surgical complications in either group. The combined RIPC and ITMB bundle may relieve ischemia–reperfusion- and pain-induced stress, as a safe and efficient means of improving renal outcomes following RALPN in patients with RCC.

## 1. Introduction

Nephron-sparing partial nephrectomy is recognized as the optimal approach for the surgical management of small renal cell carcinoma (RCC), with favorable oncological outcomes leading to increasing availability for larger and more complicated tumors despite the technical challenges, as well as better renal functional preservation without surgical complications (such as low positive surgical margin rate, hemorrhage, and urine leakage) compared to radical nephrectomy [1]. Laparoscopic partial nephrectomy, and more currently, robot-assisted laparoscopic partial nephrectomy (RALPN), have been widely adopted. RALPN shows beneficial effects with regard to the conversion rate to open surgery, warm ischemic time, kidney functional change, and length of hospitalization, attributed to the superior visualization and enhanced maneuverability with seven-degrees-of-freedom wristed devices, tremor filters, and 3D vision of the robot-assisted approach [2].

Although RALPN is expected to provide a high degree of safety and shows promising results, warm ischemia–reperfusion injury remains the single most critical modifiable factor with regard to renal function [3]. In a kidney-based study, every additional minute of warm ischemia was shown to contribute to a 6% increase in the likelihood of new-onset severe kidney impairment [4]. Thompson et al. suggested that a longer warm ischemia time (>25 min) may increase the risk of renal injury after partial nephrectomy by 2.3-fold [3]. To minimize irreversible ischemic injury, several regimens have been applied intraoperatively, including hypothermia, reducing the warm ischemic time (limited or “zero warm” ischemia), and segmental ischemia. Prolonged hilar occlusion is no longer mandatory, although partial nephrectomy has traditionally been performed with hilar clamping to provide a bloodless field and safe and efficacious surgical progress [5,6,7,8].

Various interventions have been applied to ameliorate postoperative kidney injury [9]. Remote ischemic preconditioning (RIPC) is an emerging intervention that consists of the application of brief reversible cycles of ischemia–reperfusion to an organ remote from the susceptible target organ [10]. Many groups have focused on the effects of RIPC on vulnerable vital organs, such as the kidney and heart, after major surgeries, where RIPC may trigger the release of preventive mediators from the target organ by attenuating free radical production and inflammation [11]. However, as these findings were not consistently reported by clinical trials, which showed little renoprotective effect of RIPC [12], additional interventions seem to be required to consistently achieve good renal outcomes, i.e., a bundle regimen rather than RIPC therapy alone [13]. For multimodal bundle management in various perioperative phases, well-controlled pain is a key factor in postoperative patient convalescence [14,15]. Some RIPC trials with negative renoprotective findings did not describe their pain control regimens, or the contributions thereof to analgesic outcomes during postoperative renal recovery [9,12]. In our living kidney donor study, living donors with effective analgesia consisting of intrathecal morphine block (ITMB) had lower incidences of delayed remnant kidney impairment (estimated glomerular filtration rate (eGFR) < 60 mL/min/1.73 m^2^) than those without ITMB [16]. Although the mechanism of the relation between analgesia and kidney function remains unclear, appropriate analgesia may play a role in improving pain-induced sympathetic homeostasis, and promotes safe and effective recovery of kidney function after surgery [17,18].

Therefore, we primarily investigated the effects of multimodal bundle with RIPC and ITMB on the early recovery of kidney function after RALPN. In addition, we compared the surgical and analgesic outcomes between patients with and without the bundle.

## 2. Patients and Methods

### 2.1. Ethical Considerations

**Figure 1:**
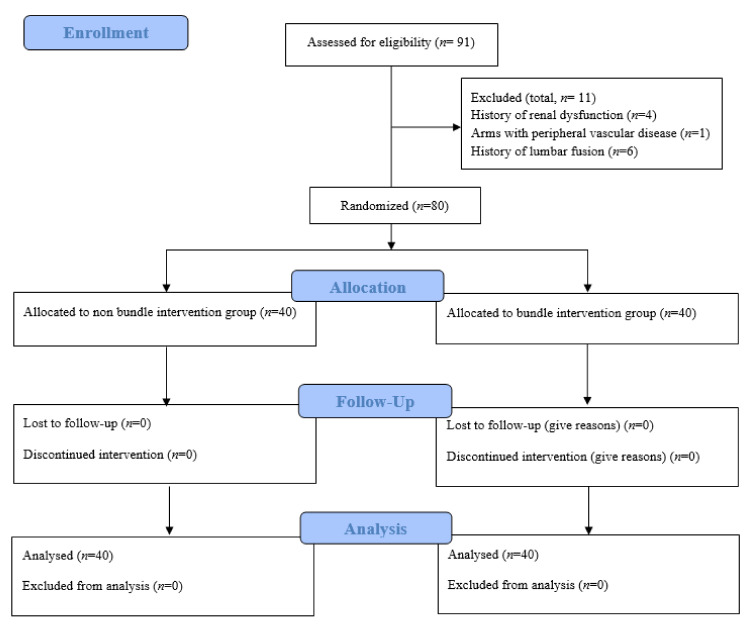
CONSORT flow diagram.

The Institutional Review Board and Ethics Committee of Seoul St. Mary’s Hospital (approval number: KC20EISI0464) approved the protocol for this single-center, prospective randomized controlled trial on 27 July 2020, which accorded with the principles of the Declaration of Helsinki. We prospectively registered the study protocol at a publicly accessible clinical registry recognized by the International Committee of Medical Journal Editors (Clinical Research Information Service, Korea; approval number: KCT0005296) on 3 August 2020. We obtained written informed consent from all patients at our hospital before enrollment in this study between 11 August 2020, and 2 March 2021. This study adhered to the Consolidated Standards of Reporting Trials (CONSORT) guidelines, and the CONSORT flow chart is presented in Figure 1 [19].

### 2.2. Study Population

The study population consisted of adult patients (19–70 years old) who made an independent clinical decision (i.e., without input from legal guardians) to undergo elective RALPN, had an RCC located in the kidney (stage I or II) [20], and were classified as American Society of Anesthesiologists (ASA) physical class I or II [21]. The exclusion criteria were a history of renal dysfunction (eGFR < 60 mL/min/1.73 m^2^ or dialysis) [22], perioperative hemodynamic instability (systolic blood pressure [SBP] < 90 mmHg; heart rate [HR] > 100 beats/min; hemoglobin < 7 g/dL; or the requirement for blood product transfusion) [23], vascular pathology in the arms preventing preconditioning in the operating room, spinal pathology precluding ITMB, the requirement for re-operation, and refusal to participate in the study.

A total of 91 patients were assessed for eligibility for inclusion in the study. Four patients had a history of renal dysfunction, one had peripheral vascular disease in the arms, and six had a history of lumbar fusion. Therefore, 80 patients were randomly divided into two groups: with (*n* = 40, bundle group) and without (*n* = 40, non-bundle group) the multimodal bundle (RIPC and ITMB; Figure 1).

### 2.3. Randomization

We randomly assigned the patients to the bundle or non-bundle group in a blinded fashion using sealed, opaque envelopes containing the group assignments (generated by a computer tool). A 1:1 ratio was applied to guarantee an equal allocation of intervention assignments across the whole study period. In the holding area where the patients waited for surgery, the attending anesthesiologists (not otherwise involved in this study) opened the topmost envelope, and the intervention was acted according to the patient’s group assignment. The attending anesthesiologists and nurses in the operating room and the attending physicians and nurses in the post-anesthesia care unit (PACU) and ward (who were not involved in further patient management or information collection, other than recording in medical forms) were aware of the group classifications.

### 2.4. RIPC and ITMB Interventions

To ensure immediate recognition of any nerve injury during ITMB, patients were not offered with sedative agents in the operating room prior to anesthesia induction. Patients in the bundle group were set in the right or left lateral decubitus position; we cleaned and draped the skin over the lumbar area with chlorhexidine, and infiltrated lidocaine (0.5 mL) to the spinal puncture site. Patients in the bundle group were infused 0.2 mg (0.2 mL) of morphine sulfate and normal saline (1 mL) between lumbar vertebrae 3 and 4 using a sterile 25 G Quincke-type spinal needle. Morphine sulfate and normal saline (total, 1.2 mL) were infused via a single injection after cerebrospinal fluid had been observed. For ITMB sham block in the non-bundle group, normal saline (0.5 mL) was used alone.

After the induction of anesthesia, and before temporary clamping of the renal artery, the bundle group took the RIPC intervention on the upper arm in the lateral position. The RIPC intervention was applied using a manual cuff inflator, which consisted of three cycles of 5-min inflation of the blood pressure cuff (to 250 mmHg, or to 50 mmHg higher than the preoperative SBP), followed by 5-min deflation of the cuff. In the non-bundle group, a blood pressure cuff was also applied on the upper arm but was not inflated.

### 2.5. RALPN and Anesthesia

**Figure 2:**
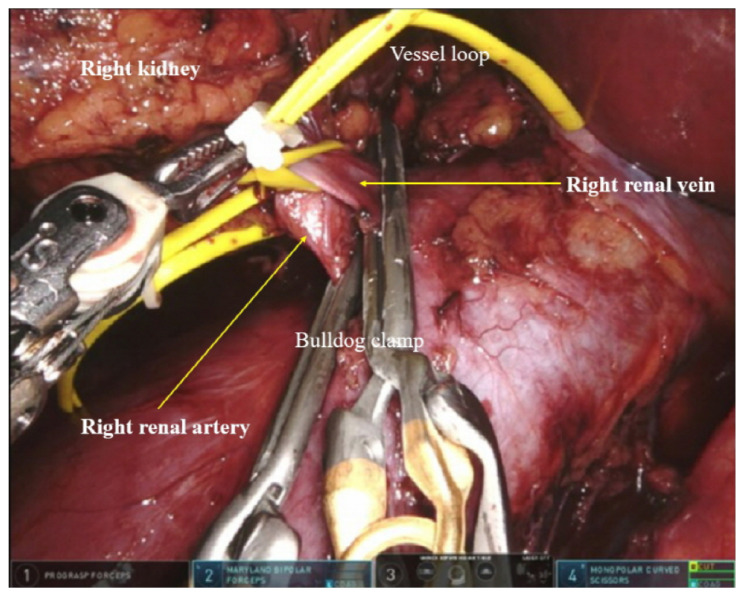
Renal vascular clamping.

Here, we briefly describe the surgical procedure and anesthetic management for RALPN. The da Vinci surgical system (Intuitive Surgical Inc., Sunnyvale, CA, USA) was applied in a three-arm configuration by expert urologists (SHH and JYL). The following robotic instruments consisted of a 30° down scope, ProGrasp forceps and Hot Shears monopolar curved scissors (both from Intuitive Surgical Inc.), permanent cautery hook, and large needle driver. After sterile draping of the surgical field with povidone, trocar insertion was performed. Under kidney traction, perirenal fat over Gerota’s fascia was dissected from mid-pole down to the inferior pole, and the hilum was exposed. Once the pedicle was had been exposed and well dissected, mannitol (0.5 mg/kg) was administered intravenously. The renal vessels were clamped carefully using laparoscopic bulldog clamps (Figure 2). Using surgical scissors, careful dissection around the tumor mass was performed to achieve negative surgical margins. Renorrhaphy was performed using the Agarwal sliding clip technique [24]. Continuous running sutures were applied to the excised kidney with a wound closure V-Loc device (Medtronic, Minneapolis, MN, USA). The collecting system and deep layer were sutured with a running 3-0 Monocryl or V-Loc suture. A double-arm suture was applied with a gold Hem-o-lok clip (Teleflex Medical, Research Triangle Park, NC, USA) using a Vicryl suture. The superficial layer was sutured with 2–3 double-arm sutures put 1 cm apart. Hem-o-lok clips were applied over the suture to press the parenchyma and maintain hemostasis. The hilar clamps were then rid. Additional Hem-o-lok clips had a locking end to ensure function. If there was any bleeding from the injured sites, the renal defect was filled with loose surgical (Ethicon Inc., Bridgewater, NJ, USA) and FloSeal (Baxter Healthcare Corporation, Freemont, CA, USA) sutures, and the end of suture sites were tied. A laparoscopic surgical bag was inserted into the abdominal cavity for tumor retrieval and then removed through the port site. Surgicel (Ethicon Endo-Surgery, Somerville, NJ, USA) and fibrin glue (Tisseel; Baxter Healthcare, Deerfield, IL, USA) were sprayed on the operation site. A single JP drain was inserted into the ascending colon flexure through the port site. The peritoneum was sutured with a Carter-Thomason port closure system (Cooper Surgical, Trumbull, CT, USA), and subcutaneous tissue and skin were sutured after meticulous bleeding control.

Balanced general anesthesia was induced with intravenous (IV) propofol (2 mg/kg) and rocuronium (0.8–1 mg/kg), followed by tracheal intubation, and was maintained with desflurane and mixed air/oxygen. Remifentanil (0.05–0.2 μg/kg/min) was IV-infused as appropriate. Multiple monitoring modalities were set, including electrocardiography, pulse oximetry, noninvasive blood pressure measurement, and bispectral index assessment. Additionally, the end-tidal carbon dioxide level and body temperature were routinely measured. At the end of surgery, neuromuscular blockade was recovered with sugammadex (4 mg/kg) under ventilation with oxygen (100%) supplementation.

All patients were provided with intravenous patient-controlled analgesia (IV-PCA), which consisted of 1000 μg of fentanyl and 0.3 mg of ramosetron. The IV-PCA program was a 1-mL bolus injection and 1-mL basal infusion, with a lockout time of 10 min. Patients suffering severe postoperative pain (pain score ≥ 7 on a numeric rating scale (NRS)) were provided rescue IV fentanyl (50 μg) for pain alleviation at the consideration of the attending physicians in the PACU and ward.

### 2.6. Primary Outcome

The eGFR, estimated using the Modification of Diet in Renal Disease formula: eGFR = 175 × standardized serum creatinine ^−1.154^ × age ^−0.203^ × 1.212 (if Black) × 0.742 (if female) [25], was measured for kidney function quantification. Serial eGFRs were measured 1 day before surgery, immediately after surgery, on postoperative days (PODs) 1 and 2, and at 3 and 6 months after surgery. The primary outcome was early recovery of remnant kidney function, determined as the lowest eGFR during the first 48 h after surgery [22,26].

### 2.7. Secondary Outcomes

Surgical complications were measured during the hospitalization period based on the Clavien–Dindo classification [27], and the length of hospital stay was investigated. Acute postoperative pain was assessed using the peak NRS during POD 2, and the severity was classified as follows: 0–3 points, mild pain; 4–6 points, moderate pain requiring non-opioid pain medication; and 7–10 points, severe pain urgently requiring opioid pain medication. In addition, the total amount of IV-PCA infused and frequency of rescue IV opioid treatment during the first 48 h postoperatively were assessed.

### 2.8. Clinical Variables

Preoperative findings included sex, age, type of RCC (site, size, and RENAL score [28]), body mass index (BMI), comorbidities, vital signs, and laboratory variables. Intraoperative findings included the total operation time, warm ischemic time, mean values of vital signs, requirement for rescue vasopressor, crystalloid input, urine output, and blood loss. Postoperative inflammation was assessed based on parameters, including the white blood cell (WBC), neutrophil, and lymphocyte counts.

### 2.9. Sample Size and Statistical Analysis

Based on data retrospectively collected from our department prior to the study, we measured the mean lowest eGFR to be 92.51 mL/min/1.73 m^2^ in the bundle group and 80.37 mL/min/1.73 m^2^ in the non-bundle group during POD 2. With a 5% risk of type 1 error, 20% risk of type 2 error, and standard deviation of 18.49 mL/min/1.73 m^2^, 37 patients would be needed in each group. Assuming a dropout rate of 10%, we designed the trial to include a total of 80 patients.

We assessed the normality of the continuous data using the Shapiro–Wilk test. Descriptive statistics for categorical factors are shown as number (proportion, %), and continuous factors are reported as the mean (standard deviation) or median (interquartile range). The *χ*^2^ test or Fisher’s exact test was applied to compare categorical factors between the two groups, while the Student’s *t*-test or Mann–Whitney *U*-test was applied to compare continuous factors as appropriate. Perioperative changes in eGFR were evaluated by repeated-measures ANOVA (RM-ANOVA) or the Friedman test with the Bonferroni *post hoc* test applied. The paired *t* test or Wilcoxon’s signed rank test was applied to compare serial eGFR measurements based on the preoperative eGFR in each group. All statistical tests were two-sided, and *p* < 0.05 was considered statistically significant. Analyses were performed using SPSS software (version 24.0; IBM Corp., Armonk, NY, USA).

## 3. Results

### 3.1. Demographic Characteristics

Our study population consisted of 37 females (46.3%) and 42 males (53.7%), with a mean age and BMI of 53 ± 11 years and 24.9 ± 3.5 kg/m^2^, respectively. All patients were ASA physical class I (35.0%) or II (65.0%). With regard to comorbidities, 21 patients had high blood pressure (HBP) (26.3%) and 14 had diabetes mellitus (DM) (17.5%). With regard to RCC findings, 36 patients had RCC on the left side (45.0%) and 44 had RCC on the right side (55%). The mean RCC size and warm ischemic time were 3.3 ± 2.1 cm and 16 ± 5 min, respectively.

Pre- and intraoperative findings were comparable between the two groups (Table 1).

### 3.2. Perioperative eGFR Outcomes

The eGFR level immediately after surgery was higher in the bundle group than non-bundle group (Table 2). In both groups, the eGFR decreased abruptly immediately after surgery and then recovered gradually over the follow-up period. The level immediately after surgery was significantly lower than the preoperative baseline in the bundle group, but serial levels on PODs 1 and 2, and at three and six months after surgery, were comparable to the baseline. In the non-bundle group, the eGFR level was lowest immediately after surgery, and serial levels on PODs 1 and 2, and at three months after surgery, remained below the preoperative baseline. The changes in eGFRs immediately after surgery, and on POD 1, were smaller in the bundle than non-bundle group.

### 3.3. Postoperative Surgical and Analgesic Outcomes

The non-bundle group showed longer hospital stays than the bundle group, but the Clavien–Dindo classification was comparable between the two groups (Table 3). There were no severe surgical (all patients were Clavien–Dindo classification I or II) or renal (no rescue renal replacement therapy) complications during the follow-up period in either group.

With regard to pain, the bundle group showed better NRS scores, a lower rescue fentanyl requirement, and a lower total IV-PCA dose than the non-bundle group.

### 3.4. Postoperative Inflammatory Outcomes

The neutrophil count, immediately after surgery and on POD 1, was lower in the bundle than in non-bundle group (Table 4). However, the WBC and lymphocyte counts on POD 2 were comparable between the groups.

## 4. Discussion

The main findings of this study were that RALPN, which is minimally invasive and has a favorable operative approach, may have benefits in terms of the preservation of renal function and minimal complications during the follow-up period in patients with RCC. However, a reduction of eGFR may be unavoidable in the early postoperative recovery phase. The intraoperative multimodal bundle regimen consisting of preemptively cycled ischemia–reperfusion episodes on nonvulnerable body parts, with good subsequent pain control, may play a key role in protecting renal function during the early recovery phase, as well as over the long term postoperatively. Our results suggested that renal functional preservation during robot-based nephron-sparing surgery may be promoted by using an intraoperative strategy to protect against ischemia–reperfusion injury and the pain-related stress induced by renal artery clamping and surgical insults.

RIPC has been attracting increasing attention as a relatively straightforward, low cost, safe, efficacious, and practical intervention to prevent acute kidney injury (AKI), which is characterized by abrupt and rapid changes in renal biochemical markers with progression to overt kidney dysfunction in hospitalized patients with acute renal failure [29]. Due to its high functional energy requirements and complicated microvascular system, the kidney is highly susceptible to ischemia–reperfusion insults, which induce reactive oxygen species production and exacerbate the inflammatory cascade of cellular responses, ultimately resulting in cell death and irreversible kidney impairment [30,31]. In animal kidney models, RIPC showed significant systemic benefits, including reducing both the area of kidney damage and exacerbation of functional surrogates associated with longer and direct ischemia–reperfusion injury [32,33]. Although the mechanisms underlying the organ protection conferred by RIPC have yet to be elucidated, neural and humoral mediators of signal transduction between the preconditioning stimulus and target organ are involved (i.e., maintenance of mitochondrial function through the opening of mitochondrial or plasma membrane adenosine-5′-triphosphate-sensitive potassium channels and closure of mitochondrial permeability transition pores), while several substances, such as adenosine and bradykinin, have been implicated in the establishment of the adverse renal environment [12,34]. Clinical human trials supported the suggestion that RIPC intervention is a promising option for vital organ protection, including within the cardiovascular and neurological systems, in patients undergoing major surgeries [35,36,37].

In kidney trials, RIPC reduced the kidney’s vulnerability to ischemia–reperfusion injury, and the favorable effects of RIPC were independent of cardiac surgical variables such as operation type, cross-clamp time, and the use of cardiopulmonary bypass [38,39,40]. In laparoscopic partial nephrectomy, the urinary retinol-binding protein level (a specific marker of proximal tubular damage) was increased to a greater extent in the control than RIPC group at POD 1 (8.4-fold vs. 3.9-fold, respectively; *p* < 0.001), and the RIPC group showed less change in GFR at one month compared to the control group (8.8% vs. 15.0%, respectively; *p* = 0.034). However, the difference in GFR had disappeared at 6 months [41]. Despite the ease of the technique, the outcomes of RIPC with regard to renal preservation are not consistent among observation periods. Chung et al. found no renal protective effect of RIPC in 81 patients undergoing partial nephrectomy. They reported no significant differences in the rate of AKI or urinary biomarker levels (urine creatinine, *β*-2 microglobulin, microalbumin, and *N*-acetyl-*β*-d-glycosaminidase) among serial analyses conducted immediately postoperatively and on PODs 1, 3, and 14 [12]. Patients undergoing partial nephrectomy show a rapid decline in postoperative global renal function due to total renal parenchymal volume reduction and ischemia–reperfusion injury of the affected remaining parenchyma. However, the rapid recovery of the remaining parenchymal structures, such as proximal tubule sites, from ischemia–reperfusion injury is one of the potential reasons for attenuation of the effect of RIPC, and prompt and proficient compensation of the healthy contralateral kidney (i.e., compensatory hypertrophy) may be the main reason for the marginal decrease seen in global renal function in the short and medium term, with *U*-shaped renal recovery seen over the long-term [9,29,42]. However, AKI, a common and serious complication of kidney surgery, is associated with increased morbidity and mortality, and the early occurrence of this renal complication should not be overlooked, particularly in the RALPN setting [43,44,45].

Surgery leads to postoperative pain, which is also a perioperative stressor; thus, the pain should be mitigated as much as possible to reduce suffering and facilitate rehabilitation, as well as to avoid complications [1]. Although the mechanism linking postoperative pain to residual kidney preservation has not been clearly elucidated, well-controlled analgesia may promote organ functional recovery, including of the kidney, with acceptable hemodynamic stability after surgery [18,46]. Our previous living kidney donor study showed that ITMB was associated with a 0.257-fold lower hazard rate for delayed functional recovery of the remnant kidney on POD 1, in association with its contribution to analgesia, and the incidences of eGFR < 60 mL/min/1.73 m^2^ on PODs 1 and 7 were lower in living donors with than without ITMB [16]. Although the neutrophil count may not be a specific inflammatory marker of surgical pain-related stress, our bundle regimen decreased the early postoperative neutrophil count, possibly reflecting mitigation of overactivity of the sympathetic stress response and subsequent improvement in organ inflammatory hemodynamics [47,48]. These findings can be considered together with the results of the present study, which indicated that a large proportion of patients in the bundle group (including ITMB) had mild and tolerable pain that required less rescue opioids on POD 2, where the decrease in eGFR was smaller compared to the non-bundle group. These findings were supported by the larger kidney transplantation study of Baar et al., in which grafts from living donors with epidural analgesia showed better renal outcomes, defined according to the application of at least one hemodialysis session on POD 7, compared to those without epidural analgesia [17]. Graft functional recovery may be dependent on a complex stress burden related to ischemia–reperfusion injury and repair cascades, which may be exacerbated by the pain-induced sympathetic activation associated with surgical insults [49,50]. Therefore, the preemptive inhibition of severe nociceptive stimuli during and after surgery may help prevent a harmful excessive sympathetic stress response, while also promoting organ microcirculation and functional recovery [51,52].

This study had some limitations. First, our primary outcome was postoperative eGFR, which is related to serum creatinine, age, and sex. However, depending on the criteria used for AKI [53], and the urinary biomarkers applied [54], renal functional performance may be measured differently from our study. Second, as our patients underwent robot-assisted nephron-sparing surgery, our findings cannot be generalized to other types of kidney surgery that may differ in the degree of invasiveness and ease of surgical access. Third, further study to analyze ITMB effectiveness for preserving renal function after RALPN is required, because the effect of ITMB alone has not yet been investigated in the context of RALPN. However, this study also had a number of strengths and showed that two efficient modifiable treatment options, namely RIPC and ITMB, can be used as renal protective regimens against ischemia–reperfusion injury and the pain-related stress response. Moreover, it showed that the two-axis bundle may be superior for renal preservation than either treatment option alone in RALPN [13].

## 5. Conclusions

A decline in global renal function is unavoidable in the setting of kidney surgery due to a loss of vascularized nephron mass and deficient recovery from ischemic insults in the operated kidney, as well as to delayed and marginal compensation in the contralateral healthy kidney. The timely and appropriate adjustment of modifiable variables related to renal progression is important for functional recovery after RALPN. Together with surgical and pharmacological methods to minimize irreversible injury, such as hypothermia, limited or zero warm ischemia, and enucleation and vascular microdissection [1], RIPC and ITMB combined relieved ischemia–reperfusion- and pain-induced stresses and appeared to be a safe and efficient means of improving renal outcomes in RALPN.

## Figures and Tables

**Table 1:** Comparison of pre- and intraoperative findings between the two groups.

Group	Non-Bundle Group	Bundle Group	*p* Value
*n*	40	40	
Preoperative Findings			
Sex (female)	15 (37.5%)	22 (55.0%)	0.178
Age (years)	49 (44–58)	58 (46–64)	0.075
Renal cell carcinoma			
Site (left kidney)	18 (45.0%)	18 (45.0%)	>0.999
Size (cm)	2.65 (1.6–4.75)	2.85 (1.8–4.7)	0.623
RENAL nephrometry scoring system (points)	6 (4–7)	6 (5–7)	0.606
Radius (maximal diameter in cm), (points)	1 (1–2)	1 (1–2)	0.679
Exophytic/endophytic properties (points)	1 (1–2)	1 (1–2)	0.932
Nearness of the tumor to the collecting system or sinus (mm), (points)	1 (1–2)	1 (1–2)	0.308
Location relative to the polar lines (points)	1 (1–2)	1 (1–2)	0.69
Body mass index (kg/m^2^)	25.35 (22.38–27.4)	24.55 (21.73–27.13)	0.444
*Comorbidity*			
Hypertension	9 (22.5%)	12 (30.0%)	0.612
Diabetes mellitus	7 (17.5%)	7 (17.5%)	>0.999
*Vital sign*			
SBP (mmHg)	121.5 (115.0–130.8)	130.5 (116.0–140.75)	0.205
DBP (mmHg)	80.0 (70.0–82.5)	73.0 (70.0–82.25)	0.455
HR (beats/min)	72.0 (66.0–80.0)	69.0 (66.0–76.0)	0.198
BT (°C)	36.45 (36.3–36.65)	36.35 (36.13–36.61)	0.316
*Laboratory values*			
WBC (×10^9^/L)	6.36 (5.52–8.26)	6.04 (4.91–7.2)	0.092
Neutrophil (%)	60.35 (50.13–68.25)	56.35 (50.7–62.33)	0.26
Lymphocyte (%)	30.3 (24.5–38.52)	33.45 (26.48–38.33)	0.361
Hemoglobin (g/dL)	14.5 (13.4–15.5)	13.7 (12.55–14.88)	0.141
Glucose (mg/dL)	104.5 (96.0–118.75)	104.0 (93.0–112.5)	0.476
Albumin (g/dL)	4.5 (4.3–4.68)	4.5 (4.33–4.8)	0.662
Total bilirubin (mg/dL)	0.75 (0.58–0.9)	0.65 (0.48–0.87)	0.204
AST (IU/L)	21.0 (18.0–27.0)	23.5 (20.0–26.0)	0.399
ALT (IU/L)	21.0 (14.25–27.75)	20.5 (15.0–31.75)	0.859
Sodium (mEq/L)	142.0 (141.0–142.75)	141.0 (140.0–142.75)	0.413
Chloride (mEq/L)	104.0 (103.0–105.0)	104.0 (103.0–105.0)	0.853
Potassium (mEq/L)	4.3 (4.1–4.48)	4.25 (4.1–4.58)	0.927
Calcium (mg/dL)	9.35 (9.13–9.58)	9.3 (9.1–9.5)	0.471
Platelet (×10^9^/L)	253.5 (212.0–294.75)	249.5 (213.75–299.25)	0.942
INR	0.99 (0.97–1.02)	0.99 (0.95–1.03)	0.421
aPTT (sec)	27.3 (25.9–28.48)	27.3 (25.88–29.25)	0.969
Intraoperative findings			
Total operation time (min)	168.5 (131.25–182.25)	150.0 (130.0–163.75)	0.069
Warm ischemic time (min)	13.46 (12.2–18.24)	14.81 (12.27–19.8)	0.637
*Average of vital sign*			
SBP (mmHg)	120.5 (112.42–125.08)	114.67 (109.42–122.33)	0.122
DBP (mmHg)	75.5 (69.83–80.92)	74.17 (69.33–80.83)	0.544
HR (beats/min)	72.0 (70.75–76.67)	69.67 (63.08–74.67)	0.052
BT (°C)	36.43 (36.28–36.63)	36.32 (36.13–36.57)	0.207
Requirement of rescue vasopressor	3 (7.5%)	3 (7.5%)	>0.999
Crystalloid input (mL/kg/h)	3.94 (3.18–5.76)	3.31 (2.34–4.78)	0.059
Urine output (mL/kg/h)	1.12 (0.43–2.19)	0.72 (0.52–1.56)	0.541
Bleeding loss (mL)	100.0 (50.0–200.0)	100.0 (50.0–100.0)	0.397

Abbreviations: RENAL nephrometry scoring system, (R)adius, (E)xophytic extent, (N)earness to the renal sinus, (A)nterior/posterior location, and (L)ocation relative to the polar lines; eGFR, estimated glomerular filtration rate; SBP, systolic blood pressure; DBP, diastolic blood pressure; HR, heart rate; BT, body temperature; WBC, white blood cell; AST, aspartate aminotransferase; ALT, alanine aminotransferase; INR, international normalized ratio; aPTT, activated partial thromboplastin time. Values are expressed as mean (±SD) and number (proportion).

**Table 2:** Comparison of the perioperative absolute estimated glomerular filtration rates, and changes therein, between the two groups.

Group	Non-Bundle Group	Bundle Group	*p* Value
*n*	40	40	
*Absolute estimated glomerular filtration rates (mL/min/1.73 m^2^)*			
Preoperative day	88.09 (80.09–96.1)	88.14 (80.04–97.96)	0.862
Immediately after surgery	71.06 (66.3–84.95) ***	83.53 (67.53–95.99) **	0.017
Postoperative day 1	77.96 (61.31–84.57) ***	83.95 (69.64–97.38)	0.072
Postoperative day 2	79.94 (67.28–90.3) **	84.99 (74.06–98.01)	0.16
3 months after surgery	85.65 (78.94–90.82) *	84.83 (78.91–98.57)	0.693
6 months after surgery	88.41 (81.59–97.88)	86.03 (80.72–104.95)	0.992
*Changes in estimated glomerular filtration rates (%)*			
Preoperative day (reference)	-	-	-
Immediately after surgery	−18.7 (−26.26–−4.23)	−9.34 (−17.35–1.78)	0.008
Postoperative day 1	−13.21 (−25.88–−2.5)	−5.14 (−16.25–12.09)	0.018
Postoperative day 2	−7.65 (−22.63–1.62)	−5.84 (−15.16–5.25)	0.24
3 months after surgery	−2.98 (−10.73–3.89)	−0.93 (−10.13–5.72)	0.57
6 months after surgery	0.63 (−10.13–7.68)	1.64 (−11.57–11.78)	0.651

* *p* < 0.05, ** *p* ≤ 0.01, *** *p* ≤ 0.001 based on the preoperative value in each group. Values are expressed as median (interquartile) and number (proportion).

**Table 3:** Comparison of postoperative surgical and analgesic outcomes between the two groups.

Group	Non-Bundle Group	Bundle Group	*p* Value
*n*	40	40	
Length of hospital stay (day)	4 (4–5)	4 (4–4)	0.032
Clavien-Dindo classification			
Grade I	38 (95.0%)	39 (97.5%)	>0.999
Grade II	2 (5.0%)	1 (2.5%)	
*Pain outcome during postoperative day 2*			
Peak numeric pain rating scale			<0.001
Mild (1–3 scale)	3 (7.5%)	24 (60.0%)	
Moderate (4–6 scale)	30 (75.0%)	15 (37.5%)	
Severe (7–10 scale)	7 (17.5%)	1 (2.5%)	
Rescue fentanyl (mcg)	50 (50–100)	0 (0–50)	<0.001
Total amount of IV-PCA (mL)	47.7 (31.6–58.7)	28.9 (15.2–36.0)	<0.001

Abbreviation: IV-PCA, intravenous patient-controlled analgesia. Values are expressed as median (interquartile) and number (proportion).

**Table 4:** Comparison of the postoperative inflammatory variables between the two groups.

Group	Non-Bundle Group	Bundle Group	*p* Value
*n*	40	40	
*White blood cell (*×*10^9^/L)*			
Immediately after surgery	12.8 (10.6–16.3)	12.5 (10.6–14.1)	0.416
Postoperative day 1	11.6 (10.1–14.6)	11.2 (9.6–12.9)	0.341
Postoperative day 2	8.9 (7.7–11.6)	8.5 (6.9–10.1)	0.092
*Neutrophil (%)*			
Immediately after surgery	85.0 (82.1–90.0)	83.1 (79.3–86.0)	0.037
Postoperative day 1	82.6 (76.7–86.9)	80.2 (77.5–82.6)	0.044
Postoperative day 2	73.7 (70.7–77.5)	72.4 (70.5–76.8)	0.583
*Lymphocyte (%)*			
Immediately after surgery	11.4 (8.8–14.2)	13.4 (10.6–15.3)	0.096
Postoperative day 1	18.2 (14.7–23.1)	20.1 (15.9–23.0)	0.361
Postoperative day 2	24.2 (19.5–30.7)	26.7 (21.2–30.7)	0.351

Values are expressed as median (interquartile) and number (proportion).

## Data Availability

The data underlying this article cannot be shared publicly to maintain the privacy of individuals that participated in the study. The data will be shared upon reasonable request to the corresponding author.
